# Characterization of preclinical Alzheimer’s disease model: spontaneous type 2 diabetic cynomolgus monkeys with systemic pro-inflammation, positive biomarkers and developing AD-like pathology

**DOI:** 10.1186/s13195-024-01416-9

**Published:** 2024-03-08

**Authors:** Xinxin Huang, Shanshan Huang, Fangyan Fu, Junzhen Song, Yuling Zhang, Feng Yue

**Affiliations:** 1 State Key Laboratory of Digital Medical Engineering, School of Biomedical Engineering, Hainan University, Sanya, 572025 China; 2 Collaborative Innovation Center of One Health, Hainan University, Hainan University, Haikou, 570228 China

**Keywords:** Alzheimer’s disease, Type 2 diabetes mellitus, Preclinical, Pro-inflammatory factor, Biomarkers, Cynomolgus monkeys

## Abstract

**Background:**

The key to the prevention and treatment of Alzheimer’s disease (AD) is to be able to predict and diagnose AD at the preclinical or early stage, but the lack of a preclinical model of AD is the critical factor that causes this problem to remain unresolved.

**Methods:**

We assessed 18 monkeys in vivo evaluation of pro-inflammatory cytokines and AD pathological biomarkers (*n* = 9 / type 2 diabetic mellitus (T2DM) group, age 20, fasting plasma glucose (FPG) ≥ 100 mg/dL, and *n* = 9 / negative control (NC) group, age 17, FPG < 100 mg/dL). Levels of pro-inflammatory cytokines and AD pathological biomarkers was measured by ELISA and Simoa Technology, respectively. 9 monkeys evaluated ex vivo for AD-like pathology (*n* = 6 / T2DM group, age 22.17, FPG ≥ 126 mg/dL, and *n* = 3 / NC group, age 14.67, FPG < 100 mg/dL). To evaluate the pathological features of AD in the brains of T2DM monkeys, we assessed the levels of Aβ, phospho-tau, and neuroinflammation using immunohistochemistry, which further confirmed the deposition of Aβ plaques by Bielschowsky’s silver, Congo red, and Thioflavin S staining. Synaptic damage and neurodegeneration were assessed by immunofluorescence.

**Results:**

We found not only increased levels of pro-inflammatory cytokines such as tumor necrosis factor-α (TNF-α) in peripheral blood (PB) and brain of T2DM monkeys but also changes in PB of AD pathological biomarkers such as decreased β-amyloid (Aβ) 42 and Aβ40 levels. Most notably, we observed AD-like pathological features in the brain of T2DM monkeys, including Aβ plaque deposition, p-tau from neuropil thread to pre-neurofibrillary tangles (NFTs), and even the appearance of extracellular NFT. Microglia were activated from a resting state to an amoeboid. Astrocytes showed marked hypertrophy and an increased number of cell bodies and protrusions. Finally, we observed impairment of the postsynaptic membrane but no neurodegeneration or neuronal death.

**Conclusions:**

Overall, T2DM monkeys showed elevated levels of peripheral and intracerebral inflammation, positive AD biomarkers in body fluids, and developing AD-like pathology in the brain, including Aβ and tau pathology, glial cell activation, and partial synaptic damage, but no neuronal degeneration or death as compared to the healthy normal group. Hereby, we consider the T2DM monkeys with elevation of the peripheral pro-inflammatory factors and positive AD biomarkers can be potentially regarded as a preclinical AD model.

**Supplementary Information:**

The online version contains supplementary material available at 10.1186/s13195-024-01416-9.

## Introduction

Alzheimer’s disease (AD) is the most common neurodegenerative disorder of dementia. About 50 million dementia patients are estimated worldwide, and with a continuous increase in average lifespan, this number will reach 152 million by 2050 [1]. To date, the pathogenesis of AD is unknown, leading to a lack of effective treatments. Substantial evidence supports the observation that AD is an insidiously progressive disease, and neuropathologic changes accrue in the brain 10–30 years before the onset of clinical symptoms, representing a preclinical phase [2]. This has also been demonstrated in post-mortem studies in a substantial number of cognitively normal older adults [3–5]. In the past few years, an increasing number of studies have focused on the preclinical stage and attempted prediction through biomarkers of AD pathology. For example, cerebrospinal fluid (CSF) Aβ42 levels in cognitively normal individuals are negatively correlated with amyloid imaging markers that predict progression to symptomatic Alzheimer’s disease and brain atrophy [6–9]. In autosomal dominant AD patients, Aβ 42 is decreased, and total tau (t-tau) and tau protein phosphorylation (p-tau181) levels are increased, decades earlier than the expected onset of symptoms [10–12]. However, few preclinical Alzheimer’s disease studies have validated the relationship between biomarkers and the gold standard diagnosis of neuropathology to assess diagnostic accuracy. On the clinical side, the only clear pre-symptomatic stage of dementia in routine diagnostic tests is mild cognitive impairment (MCI), and there is no common definition or research vehicle available for preclinical AD.

AD is increasingly recognized as a complex multifactorial disease given the interconnection of the genetic component with other risk factors (e.g., co-morbidities, vascular risk factors, environmental, and lifestyle factors) [13]. Recent studies have shown that type 2 diabetes mellitus (T2DM) is a systemic chronic inflammation [14–16]. AD is known as type 3 diabetes [17–20]. Epidemiological studies have shown that the risk of developing AD in diabetes is 1.25–1.91 times higher [21]. Similarly, several in vivo studies support that T2DM promotes AD pathology. For example, in T2DM pathology, islet amyloid polypeptide was detected in Aβ plaques in the brains of patients with both AD and T2DM, suggesting its potential interaction with Aβ to promote AD pathology [22]. Accumulating evidence indicates DM-accelerated Aβ pathology in nonhuman primate brains [23]. Similarly, T2DM increases cognitive impairment and accelerates AD pathology in rodents [24–27]. These studies have shown that AD and T2DM share many pathophysiological mechanisms and may be a vehicle for AD.

In this study, we aimed to detect pro-inflammatory cytokines and AD biomarkers in the T2DM monkeys and compare with healthy monkeys. On this basis, AD-like pathology changes were evaluated in the brains of T2DM monkeys. On the one hand, it can provide an applicable model and its screening basis for preclinical AD research. At the same time, it provides primary data for biomarkers to detect AD neuropathological changes reliably.

## Materials and methods

### Animals

This study included 27 cynomolgus monkeys (*Macaca fascicularis*) divided into AD biomarker and histopathology cohorts. In the AD biomarker cohort, 18 cynomolgus monkeys (*n* = 9 / T2DM group, age 20, and *n* = 9 / negative control group, age 17) were used to analyze biomarkers and inflammation factors. In the histopathology cohort, 9 cynomolgus monkeys (*n* = 6 / T2DM group, age 22.17, and *n* = 3 / NC group, age 14.67) were used for AD-like pathology. Specific information is shown in Supplementary Table 1.

The animals were screened as follows: (1) veterinarians and researchers refer to non-human primate diagnostic criteria for T2DM [28] and screen T2DM cynomolgus monkeys by repeating the fasting plasma glucose (FPG) level, including FPG ≥ 100 mg/dL in the T2DM group and FPG < 100 mg/dL in the healthy control group; (2) no history of neurological or psychiatric diagnosis; (3) no history of drug/alcohol abuse; (4) not currently taking any psychoactive drugs, nutrient supplements affecting glucose tolerance within 3 months of participation in any other study, and oral contraceptives, acetylsalicylic acid, steroids, protease inhibitors, and so forth; and (5) no contraindications to MRI scanning.

Before starting the experiment, all animals had never been involved in any pharmacological tests or studies. During the study period, the animals were kept in stainless steel monkey cages in the nonhuman primate facility of Thinxon Biomedical Co. Ltd., which obtained the laboratory animal use license accredited by Guangxi province. The animals were fed twice daily, supplemented with fresh fruit, water libitum, and various enrichment foods once a day. All animals were maintained under 12-h light and 12-h dark cycles at room temperature 22–28 °C with a relative humidity of 30–75%. In addition, animals underwent a thorough examination before the experiments and were confirmed to be free of other diseases such as tuberculosis. All animal experiments were approved by the Institutional Animal Care and Use Committee.

### Peripheral blood (PB) and cerebrospinal fluid (CSF) collection

PB and CSF samples were collected from the monkeys in the AD biomarker cohort. In short, following standard procedures animals were anesthetized with intramuscular injection of atropine and tiletamine hydrochloride and zolazepam hydrochloride (Telazol^®^). PB was collected via in an antecubital vein in the arm in appropriate EDTA-K_2_ and SST tubes (BD, USA) from all animals. CSF was collected aseptically by lumbar puncture from all animals. All samples were divided into aliquots and frozen at -80 °C.

### Pro-inflammatory cytokines measurements in serum

Serum samples were analyzed for Interleukin 1beta (IL-1β), Interleukin-6 (IL-6), and tumor necrosis factor-α (TNF-α) using commercially available enzyme-linked immunosorbent assay (ELISA) kits (Abcam, USA; ab178013, ab214025, and ab181421, respectively).

### AD pathological biomarkers measurements in plasma and CSF

Plasma and CSF were collected and β-Amyloid 1–42 (Aβ42), β-Amyloid 1–40 (Aβ40), neurofilament light chain (NfL), and glial fibrillary acidic protein (GFAP) concentrations were measured by Neurology 4-plex E Kit (Cat. No. 103,670) with sensitive Single molecule array (Simoa) technique (Quanterix Corp., Billerica, MA, USA). And p-tau-181 was measured by p-tau-181 Advantage V2 Kit (Cat. No. 103,714) assessed with Simoa technology.

### Immunohistochemical analysis

Isolated fresh brains were sliced into 4-mm thick tissue sections using the coronal plane as a reference and fixed in 4% paraformaldehyde for 72 h. Then, the fixed brain tissues were sequentially dehydrated in 15–30% sucrose solution, sliced further into 40-µm thick sections using a SM2000R microtome (Leica, Germany), and stored in ethylene glycol at -20 °C.

Subsequently, the sections were subjected to immunohistochemical staining. The primary and secondary antibodies used in this study were listed in Supplementary Table 2. Briefly, hydrogen peroxide was used to quench the activity of endogenous peroxidase. Citrate buffer was used for antigen repair. The sections were incubated with primary antibodies at 4°C for 16 h and then with horseradish peroxidase (HRP) -labeled secondary antibodies. The avidin-biotin-peroxidase complex (prepared from VECTASTAIN ABC Reagent kit) and substrate chromogen 3,3’-diaminobenzidine tetrahydrochloride hydrate (DAB) were used to visualize the immune complex. The whole brain images were captured under a VS200 Virtual Slide Microscope (Olympus, Japan).

### Modified bielschowsky’s silver staining

The ammoniacal silver solution, developer, fixed liquid, and ammonia water were prepared according to the manufacturer’s instructions. 40-µm thick free-floating brain sections were mounted on slides and rehydrated in a graded ethanol series. Sections were immersed in a silver nitrate solution at 37 °C until golden brown. Then, sections were immersed in ammoniacal silver solution until the tissue turned brown. Finally, sections were immersed in the developer until the tissue turned dark brown, and the slides were sealed with neutral resin. Slides were scanned by a scanning microscopy imaging system (VS200 Virtual Slide Microscope, Olympus, Japan).

### Thioflavin S staining

40-µm thick free-floating brain sections were placed in 0.025% thioflavin S (solubilized in 50% ethanol) and incubated at room temperature for 30 min. The sections were decolorized in 50% ethanol, co-stained with DAPI, and sealed with an antifade mounting medium. Sections were scanned by a scanning microscopy imaging system (VS200 Virtual Slide Microscope, Olympus, Japan).

### Congo red staining

40-µm thick free-floating brain sections were incubated in Congo red staining to visualize amyloid deposits and washed in an alkaline ethanol differentiation buffer containing 80% ethanol and 0.2% potassium hydroxide. Finally, the sections were stained with hematoxylin and sealed with neutral resin. Slides were scanned by a scanning microscopy imaging system (VS200 Virtual Slide Microscope, Olympus, Japan).

### Immunofluorescence analysis

Immunofluorescences identified the co-localization of AD-like pathology. Briefly, the sections were incubated with primary antibodies derived from different host species at 4 °C, followed by incubation with species-appropriate Alexa Fluor® 488-, Alexa Fluor® 555-, and Alexa Fluor® 594-labeled secondary antibodies (Abcam, Cambridge, UK) for 2 h at room temperature (see supplemental Table 2). Subsequently, the sections were incubated with an autofluorescence quencher and DAPI for nuclear counterstaining. The images of immunofluorescence staining were acquired with a VS200 Virtual Slide Microscope.

### Western blot

Total protein was extracted from brain tissues. The protein concentration was measured using an Enhanced BCA Protein Assay Kit (Servicebio Biotechnology, China). Proteins were diluted with 5× sodium salt/polyacrylamide gel electrophoresis (SDS/PAGE) sample loading buffer. Afterwards, protein samples were loaded on SDS/PAGE gels, separated, and transferred to polyvinylidene fluoride (PVDF) membranes (Millipore, Billerica, MA, USA). Then, the blots were incubated with the following primary antibodies overnight at 4 ℃ and incubated with secondary antibodies for 2 h at room temperature (see supplemental Table 2).The membranes were scanned using the Imaging System (ChemiSciope6000, CLINX, China).

### Fluoro-Jade C (FJC) staining

The degenerating neuron stain kit (Solar bio, Beijing, China), which labels Fluoro-Jade (FJC) positive neurons was used to visualize degenerating neurons in 40-µm thick sections. Briefly, brain sections were incubated at room temperature using reagents diluted in distilled water, sealed with neutral resin and view with the aid of a VS200 Virtual Slide Microscope.

### Statistical analysis

Results are presented as mean ± SD. For detection of statistically significant differences in the results, data were analyzed with unpaired t-test of GraphPad Prism (GraphPad, San Diego, CA, USA). Significant p-values are defined as ns *p* > 0.05, *p ≤ 0.05, **p ≤ 0.01, and ***p ≤ 0.001.

## Results

### Inflammatory factors in the T2DM monkeys

**Fig. 1:**
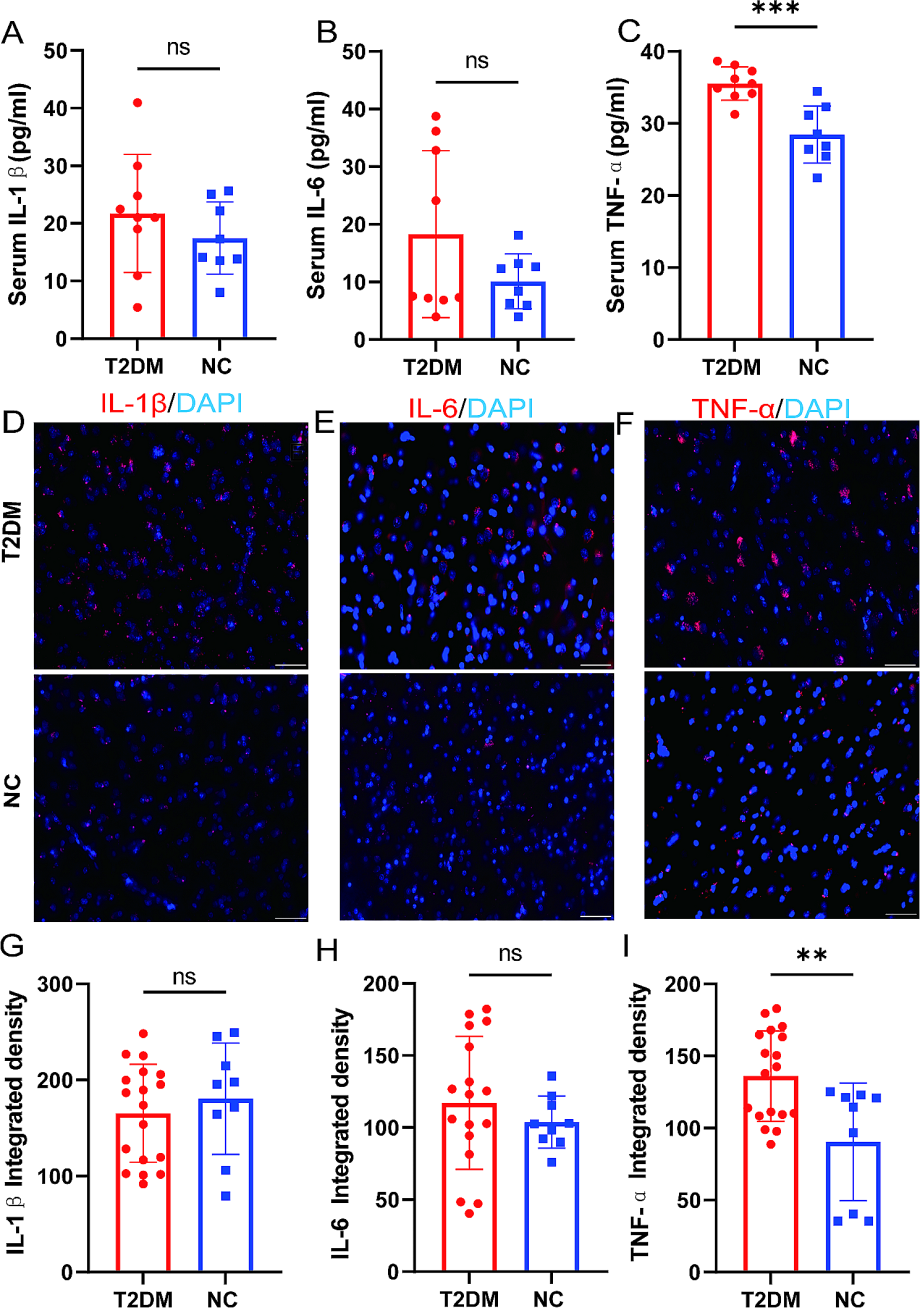
The expression levels of three inflammatory factors in T2DM monkeys. (**A**) The levels of serum IL-1β of the T2DM group and NC group. (**B**) The levels of serum IL-6 of the T2DM group and NC group. (**C**) The levels of serum TNF-α of the T2DM group and NC group. (**D, G**) The expression levels of IL-1β in the brain of T2DM group and NC group. (**E, H**) The expression levels of IL-6 in the brain of T2DM group and NC group. (**F, I**) The expression levels of TNF-α in the brain of T2DM group and NC group. Results presented as mean ± SD. Unpaired t-test was applied (*n* = 9/T2DM group; *n* = 9/NC group) (*p* > 0.05 = ns; *p* ≤ 0.05 = *; *p* ≤ 0.01 = **; *p* ≤ 0.001 = ***). Scale bars: 50 μm (*n* = 6/T2DM group; *n* = 3/NC group)

Among the three inflammatory factors tested (IL-1β, IL-6, and TNF-α), only TNF-α showed differences between the groups (*p* < 0.001); the TNF-α concentration in the T2DM group was higher than that in the NC group (Fig. 1C). The level of IL-1β and IL-6 were higher in the T2DM group than in the NC group, but there was no significant difference (Fig. 1A-B) (*p* > 0.05). Similarly, we also observed the expression level of IL-6, IL-1β, and TNF-α in the brains of the T2DM group and NC group. The results showed that only TNF-α expression was increased in T2DM than in the NC group (Fig. 1D–I).

### AD pathological biomarkers in T2DM monkeys

**Fig. 2:**
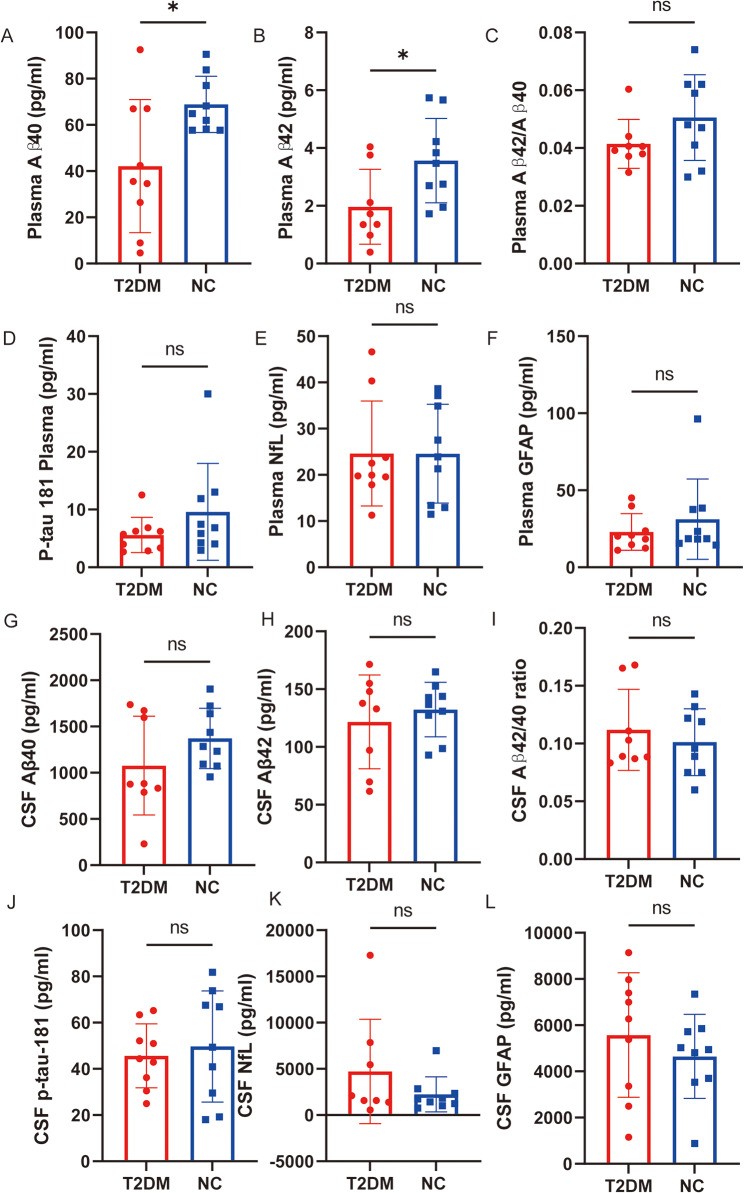
Levels of AD pathological biomarkers in T2DM monkeys. **A, G** The levels of plasma (**A**) and CSF (**G**) Aβ40 of the T2DM group and NC group. **B, H** The levels of plasma (**B**) and CSF (**H**)Aβ42 of the T2DM group and NC group. **C, I** The levels of plasma (**C**) and CSF (**I**) Aβ 42/40 ratio of the T2DM group and NC group. **D, J** The levels of plasma (**D**) and CSF (**J**) p-tau 181 of the T2DM group and NC group. **E, K** The levels of plasma (**E**) and CSF (**K**) neurofilament protein light chain (NfL) of the T2DM group and NC group. **F, L** The levels of plasma (**F**) and CSF (**L**) GFAP of the T2DM group and NC group. Results presented as mean ± SD. Unpaired t-test were applied (*n* = 9/T2DM group; *n* = 9/NC group) (*p* > 0.05 = ns; *p* ≤ 0.05 = *; *p* ≤ 0.01 = **; *p* ≤ 0.001 = ***)

The plasma Aβ42 and Aβ40 in the T2DM group were significantly lower than that in the NC group (*p* < 0.05) (Fig. 2A, B). But there was no significant difference in the expression level of plasma Aβ40/Aβ42 ratio between the two groups (*p* > 0.05) (Fig. 2C). The CSF Aβ40 was lower in the T2DM than in the NC group, but there was no significant difference (*p* > 0.05) (Fig. 2G).

### Aβ plaques developed in the brains of T2DM monkeys

**Fig. 3:**
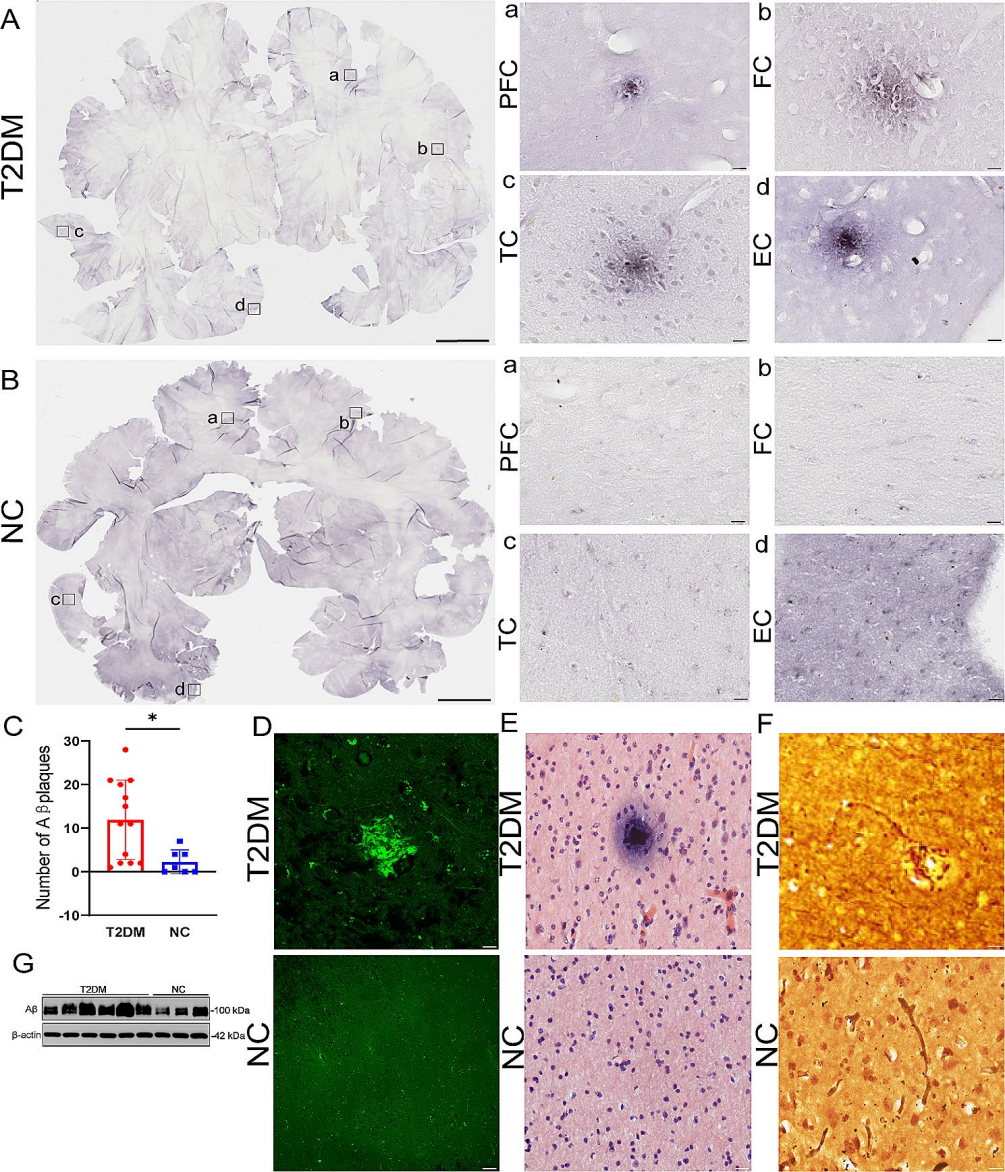
Histological staining of Aβ pathologic features in the brain of T2DM monkeys. **A-B** Images of DAB staining of the brain of representative symptomatic T2DM group (**A**) and NC group (**B**) showing 6E10 immunoreactivity. High-magnification images show Aβ plaques in different brain regions on the right. **C** Quantification of Aβ plaques in the brain of T2DM group and NC group. **D** The detection of Aβ plaques by Thioflavin S staining. **E** The detection of Aβ plaques by Congo red staining. **F** The histological visualization of Aβ plaques by silver staining. **G** Levels of Aβ in the cortex of monkeys were measured using Western blot analysis. β-Actin was used as a loading control. Data are represented as mean ± SD. Statistical differences are evaluated with a two-tailed unpaired Student’s t-test (*n* = 6/T2DM group; *n* = 3/NC group) (*p* > 0.05 = ns; *p* ≤ 0.05 = *; *p* ≤ 0.01 = **; *p* ≤ 0.001 = ***). Scale bars: 5 mm (A, B), 50 μm (**A, B** a–d, **D-F**).

By scanning whole brain sections, we observed immunopositivity for 6E10 in T2DM monkeys and distributed in massive regions, including the prefrontal cortex (PFC), frontal cortex (FC), temporal cortex (TC), and entorhinal cortex (EC) (Fig. 3A). The identified plaques exhibited dense or diffuse characteristics, akin to the typical Aβ plaques found in the brains of AD patients [29]. In contrast, healthy controls either did not find Aβ plaques or presented deposits confined to limited regions or produced intracellularly (Fig. 3B). In quantitative analysis, we quantified the presence of Aβ plaques number in all regions. The results revealed a significant increase in the number of Aβ plaques in the T2DM group compared to the NC group (Fig. 3C). In addition, the Aβ plaques in the T2DM monkey brain were recognizable by other standard measurements, including Thioflavin S (Fig. 3D), Congo Red (Fig. 3E), and silver staining (Fig. 3F). The results of Western blot assay were consistent with these findings, we observed more high molecular weight Aβ in the T2DM group compared to the NC group (Fig. 3G). Collectively, these findings suggest that T2DM may contribute to the deposition of Aβ in the brain.

### NFTs in the brain of T2DM monkeys

**Fig. 4:**
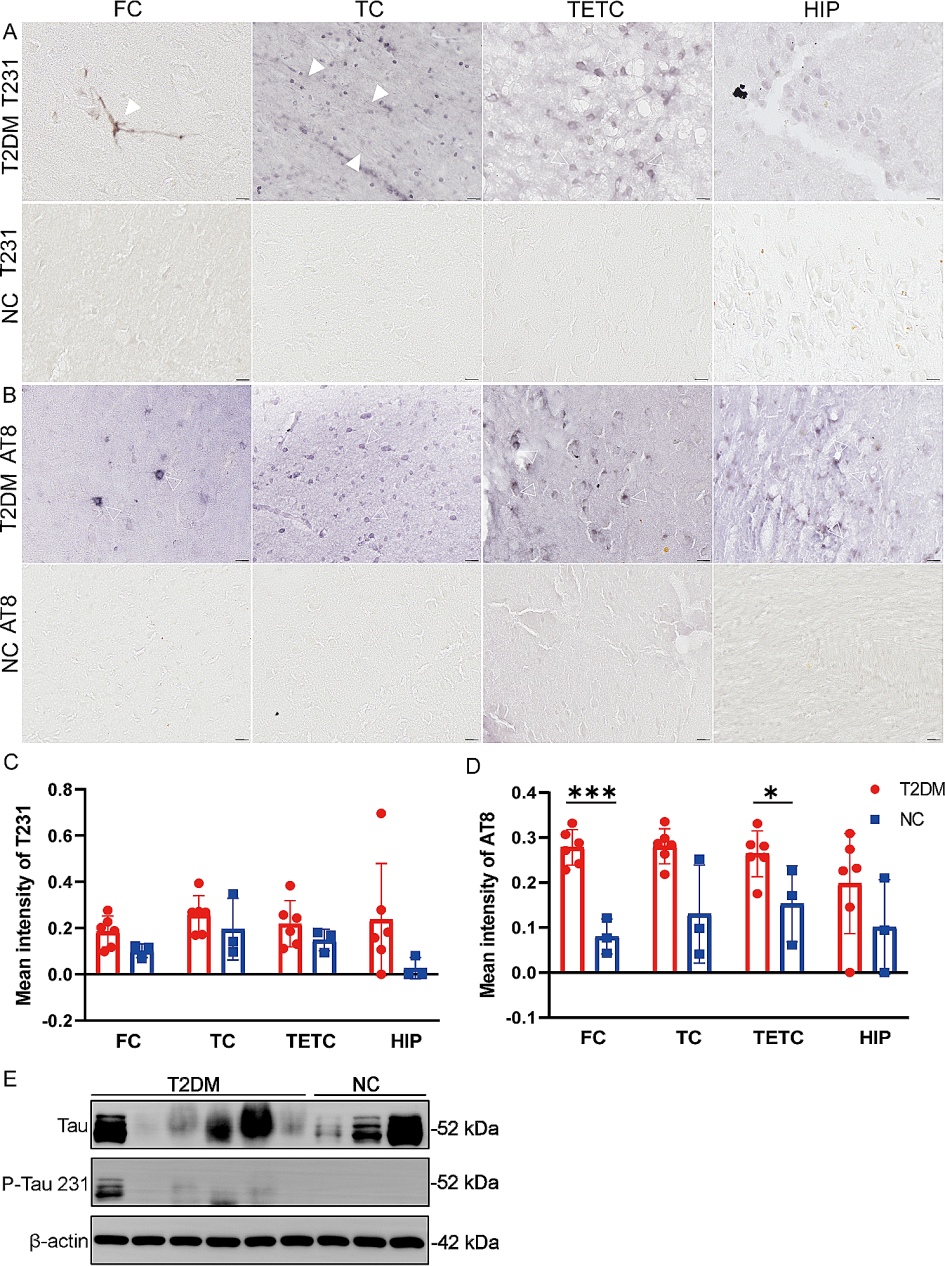
Histological staining of NFT pathological features in the brain of T2DM monkeys. **A-B** Images of DAB staining in the FC, TC, TETC, and HIP of representative symptomatic T2DM group (**A**) and NC group (**B**) showing p-tau immunoreactivity. **C-D** Quantification of p-tau in the brain of the T2DM group and NC group. **E** Levels of p-tau 231 in the cortex of monkeys were measured using Western blot analysis. β-Actin was used as a loading control. Data are represented as mean ± SD. Statistical differences are evaluated with a two-tailed unpaired Student’s t-test (*n* = 6/T2DM group; *n* = 3/NC group) (*p* > 0.05 = ns; *p* ≤ 0.05 = *; *p* ≤ 0.01 = **; *p* ≤ 0.001 = ***). The arrowheads indicate neuropil threads and empty arrowheads indicate droplet-like inclusions. Scale bars: 50 μm

We employed site-specific phosphorylated tau (p-tau) antibodies to identify neuropathological changes associated with neurofibrillary tangles. Typical pre-NFTs of Threonine 231 (p-tau T231) immunostaining identified neuropil threads staining in the FC and TC (arrowheads) and granular homogenous staining in the soma of transentorhinal (TETC), suggestive of pre-tangles in the T2DM monkeys (Fig. 4A). Compared to p-tau T231, serine202/threonine 205 (p-tau AT8) showed eNFT immunopositivity neurons in the FC, TC, and HIP of brain in T2DM monkeys. However, a few extracellular-NFTs (eNFTs, arrows) were also observed, characterized as empty within the neuron (Fig. 4B). And quantitative analysis showed that the AT8 expression levels in the brains of the T2DM group and NC group were significantly increased in FC and TETC (Fig. 4C, D). The results of Western blot assay were consistent with these findings, as the levels of p-Tau in the cortex were increased in T 2DM monkeys compared with NC monkeys (Fig. 4E).

### Activated microglia and astrocytes in the brain of T2DM monkeys

**Fig. 5:**
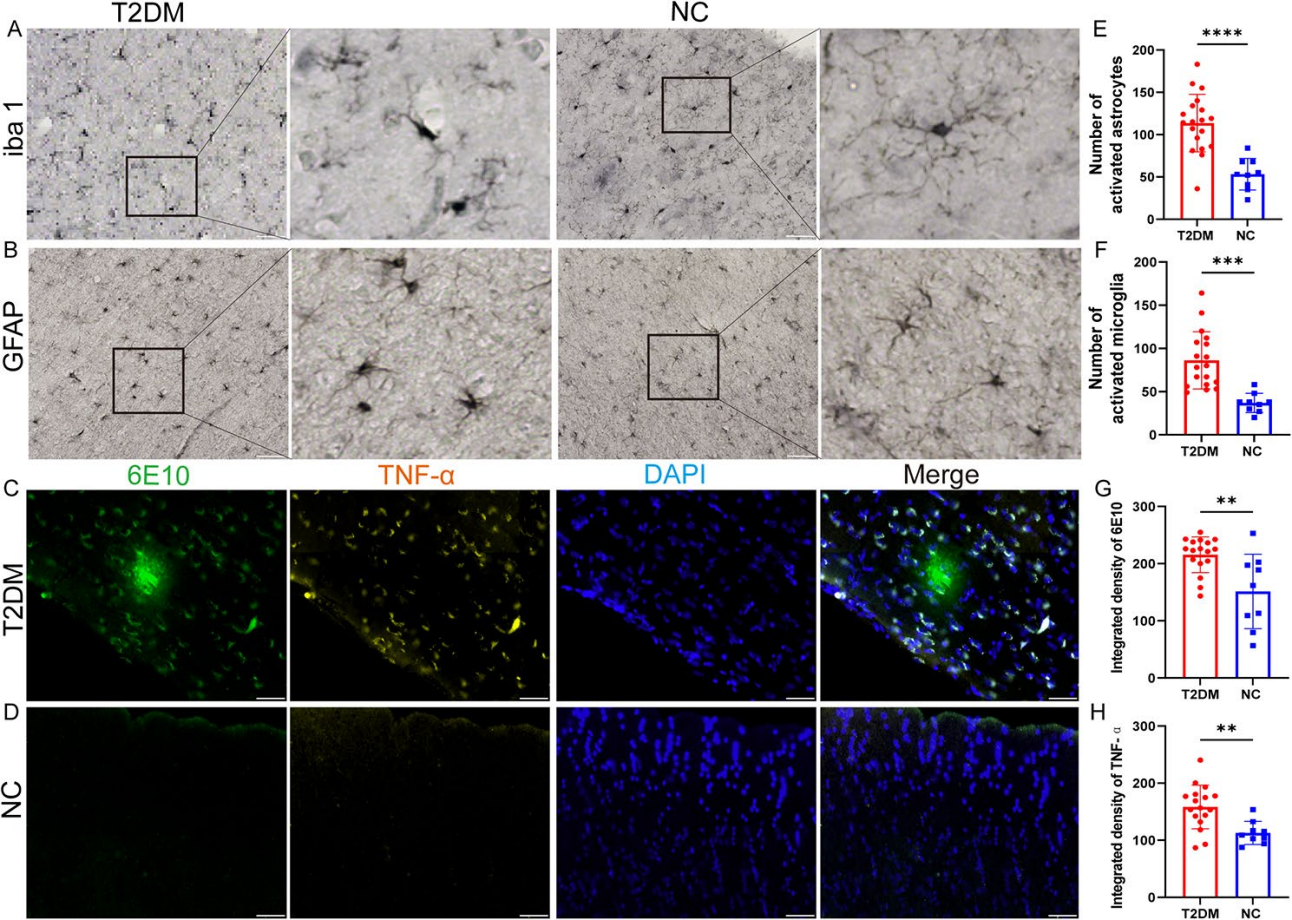
Histological staining of microglia and astrocytes in the brain of T2DM monkeys. **A** Representative image of monkey brains immunostaining for microglial cells. High-magnification images are shown on the right. **B** Representative image of monkey brains immunostaining for astrocyte cells. High-magnification images are shown on the right. **C** Merge image of Aβ plaques (6E10) immunofluorescence staining (green) and TNF-α (yellow) in the T2DM monkeys. **D** Merge image of Aβ plaques (6E10) immunofluorescence staining (green) and TNF-α (yellow) in the NC group. **E** Number of activated microglia in the T2DM group and NC group. **F** Number of activated astrocytes in the T2DM group and NC group. **G** Quantification of mean intensity of 6E10 in the T2DM group and NC group. **H** Quantification of mean intensity of TNF-α in the T2DM group and NC group. Results presented as mean ± SD. Unpaired t-test were applied (*n* = 9/T2DM group; *n* = 3/NC group) (*p* > 0.05 = ns; *p* ≤ 0.05 = *; *p* ≤ 0.01 = **; *p* ≤ 0.001 = ***). Scale bars represent 50 μm

Microglia states can be classified as functional as resting (branching-like) and activated (amoeboid-like). The microglia can be measured through the quantification of iba-1. In the brain of NC group, iba1-stained resting microglia were characterized by intact cell bodies and abundant dendrites (Fig. 5A). But in T2DM monkeys, iba1-stained microglia displayed a rounded or rod-like shape with deformed or reduced dendritic branching (Fig. 5A). We also find significantly increase of activated microglia level in the T2DM monkeys than in the NC group (Fig. 5C). Similarly, astrocytes can be measured through quantification of GFAP. Compared with the NC group, GFAP-positive astrocytes were lightly immune reactive in dendritic processes in the brains of T2DM monkeys (Fig. 5B). Consistent with this observation, we found that GFAP-positive astrocytes also significantly increased in the T2DM monkeys than in the NC group (Fig. 5D). In addition, we also validated the periphery Aβ-positive biomarkers and elevated TNF-α factors found in the brain of T2DM monkeys. The results showed that TNF-α was co-expressed near Aβ plaques in T2DM monkeys (Fig. 5C), and both Aβ plaques and TNF-a fluorescence mean intensity were significantly increased in the T2DM group compared with the NC group (Fig. 5H-I).

### Synaptic expression in the cerebral cortex of T2DM monkeys

**Fig. 6:**
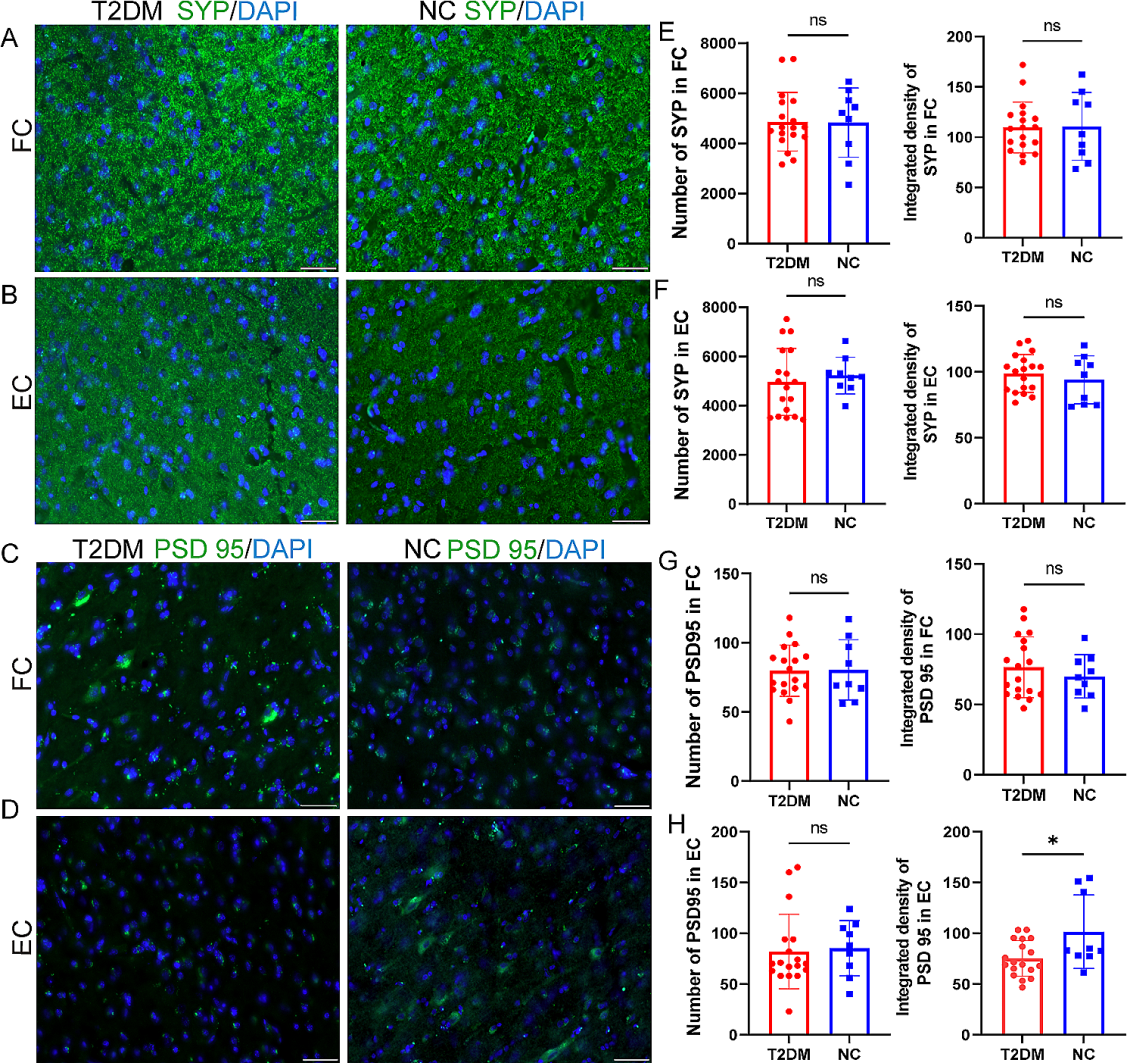
The synaptic damage in the brain of T2DM monkeys. **A-B** Synaptophysin (SYP) staining (green) of the FC (**A**) and EC (**B**) in the T2DM group and NC group. **C-D** PSD95 staining (green) of the FC (**C**) and EC (**D**) in the T2DM group and NC group. **E-F** Quantification of number and mean intensity of SYP in FC (**E**) and EC (**F**)of the T2DM group and NC group. **G-H** Quantification of number and mean intensity of PSD95 in FC (**G**) and EC (**H)** of the T2DM group and NC group. Results presented as mean ± SD. Unpaired t-test were applied (*n* = 9/T2DM group; *n* = 3/NC group) (*p* > 0.05 = ns; *p* ≤ 0.05 = *; *p* ≤ 0.01 = **; *p* ≤ 0.001 = ***). Scale bars represent 50 μm

Prolonged activation of inflammatory factors can lead to damage to neurons and synapses in the brain [30]. To explore this concept, we next evaluated the expression of the presynaptic membrane (synaptophysin, SYP) and postsynaptic membrane (postsynaptic density-95, PSD95) in the brains of monkeys. The results indicated that the number and intensity of SYP were not significantly decreased in the T2DM group (Fig. 6A-B, E-F). However, the postsynaptic membrane displayed a decrease in the PSD95 immunoreactivity expression of EC in T2DM groups (Fig. 6H).

### Neurodegeneration and neuron expression in the brain of T2DM monkeys

**Fig. 7:**
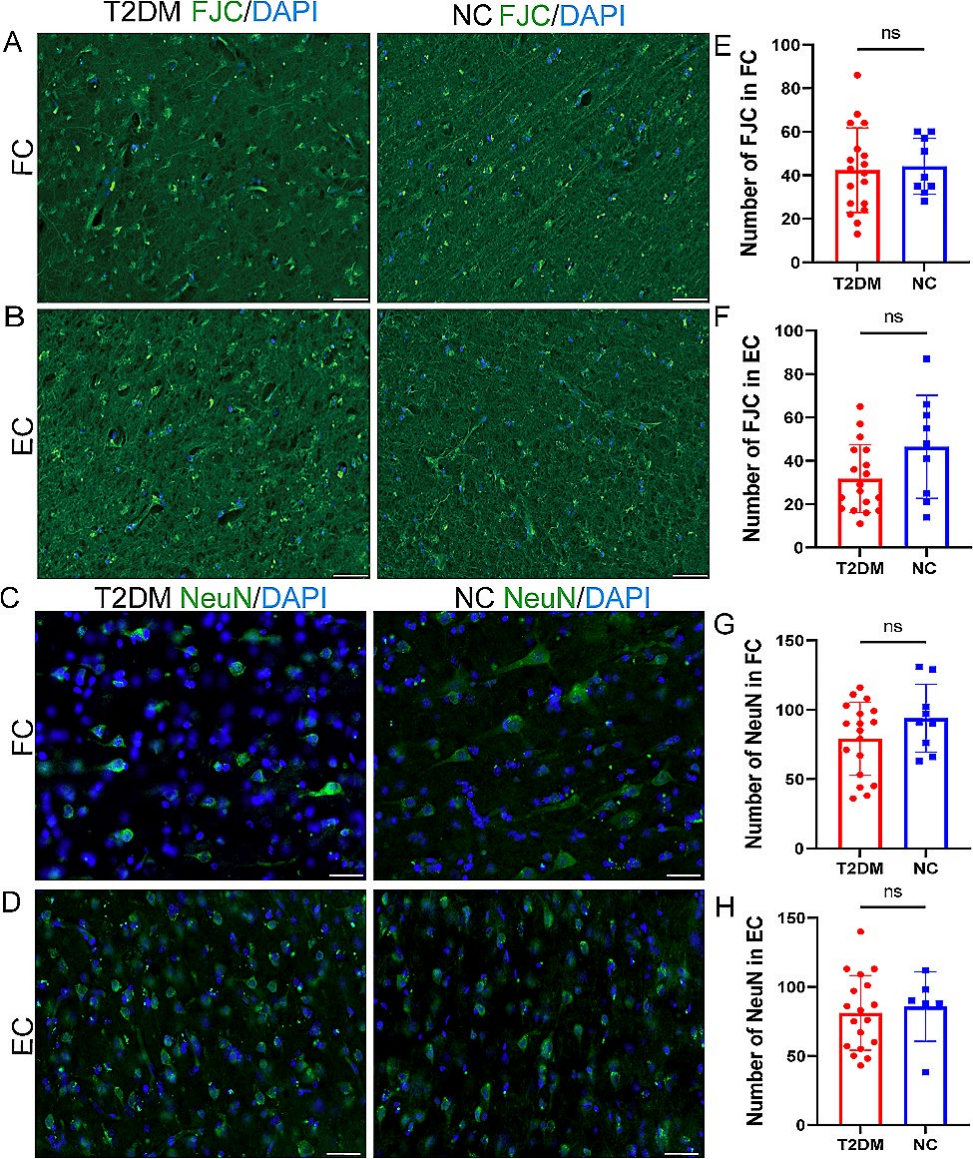
The neurodegeneration and neuron expression in the brain of T2DM monkeys. **A-B** FJC staining (green) of the FC (**A**) and EC (**B**) in the T2DM group with NC group. **C-D** NeuN (green) of the FC (**C**)and EC (**D**) in the T2DM group with NC group. **E-F** Quantification of FJC number in FC (**E**) and EC (**F**) of T2DM group and NC group. **G-H** Quantification of NeuN numbers in FC (**G**) and EC (**H**) of the T2DM group with NC group. Results presented as mean ± SD. Unpaired t-test were applied (*n* = 6/T2DM group; *n* = 3/NC group) (*p* > 0.05 = ns; *p* ≤ 0.05 = *; *p* ≤ 0.01 = **; *p* ≤ 0.001 = ***). Scale bars represent 50 μm

Finally, we studied the brain to measure markers of neurodegeneration and neuronal nucleus. We measured neurodegeneration (FJC), and neuronal nucleus (NeuN) in the T2DM group and NC groups. There was no evidence of a reduction in any of the neurodegeneration in the T2DM group (Fig. 7). Likewise, the neuronal nucleus was unchanged in the T2DM group compared to the NC group in the FC and EC. Together, these results provide strong evidence that T2DM accelerates AD pathophysiologic processes but does not produce significant synaptic dysfunction and/or neurodegeneration.

## Discussion

Although a few studies have reported the preclinical AD pathophysiology and pathogenetic features, there still needs to be more preclinical AD models or research vectors. In this study, we found that Aβ-positive biomarkers and an increase of pro-inflammatory factor TNF-α in peripheral T2DM monkeys may serve as preclinical AD-related research vehicles.

According to the NIA-AA Research Framework, plasma Aβ42 levels can be used to assess Aβ plaque formation in the brain [31–33]. A study used the Simoa method to measure plasma AD biomarkers in a population clinically diagnosed with AD, found differences in the level of Aβ42 and Aβ42/Aβ40 ratio, which is consistent with our findings [34, 35]. Likewise, we found similar changes in CSF, only without a statistically significant difference. This is understandable because we detected for AD biomarkers in the T2DM disease state, which has not yet reached AD. Plasma and CSF p-tau were not altered in the T2DM group compared to the NC group. Previous studies have shown the diagnostic value of plasma p-tau, but only in the stage of full-blown dementia [31, 36]. In addition, we also found higher NfL and GFAP levels in the CSF of T2DM group than in the NC group. Unlike Aβ or p-tau, NfL and GFAP are not associated with a specific neuropathologic process, so they can only be used as auxiliary indicators. However, because of the relatively low number of samples analyzed and the lack of a definitive explanation for the discrepancy between CSF and plasma, additional studies in larger cohorts should be conducted to investigate the consistency of these results. Overall, we found Aβ decrease in the periphery of T2DM monkeys, consistent with stage 1 of the NIA-AA for preclinical AD, suggesting that T2DM monkeys may be in the preclinical AD stage.

We all know that a biologically complex neurodegenerative disease such as AD is unlikely to be caused by a single pathogenic event, and the finding of a panel of plasma biomarkers characterizing AD pathology, rather than a single marker, was to be expected. Preclinical studies provide evidence that systemic inflammation may be associated with AD pathogenesis, in which Aβ and neuroinflammation arise due to a direct response to systemic inflammation and part of the brain’s innate immune response to inflammation [37–41]. Individuals with increased levels of proinflammatory cytokines (IL-1, IL-6, and TNF-α) also have a higher risk of developing AD [42, 43]. Among these cytokines, TNF-α is one of the most well-characterized cytokines in the pathogenesis of AD, as TNF-α levels are strongly associated with cognitive decline, neuronal toxicity, and cerebral apoptosis [44, 45]. In the present study, elevated levels of inflammatory factors TNF-α were observed in the periphery and brain of T2DM monkeys, consistent with AD patients [46–48]. Although the use of inflammatory mediators as peripheral AD biomarkers has not been established, we found abnormalities in peripheral levels of both Aβ and TNF-α in T2DM monkeys, so we hypothesized that a combination of Aβ and TNF-α might more accurately identify T2DM monkeys in a preclinical AD state.

Most importantly, we found Aβ plaques deposition in the brain of T2DM monkeys, including the PFC, FC, TC, and EC, and the morphology of Aβ plaques was similar to the pathology in the brains of AD patients [29]. NFT is another typical feature of AD pathology. Among them, antibodies to p-tau T231 and AT8 are widely used in AD-related studies to label different forms and stages of NFTs. As expected, we observed p-tau T231 and AT8-labeled positive neurons with diffuse granular cytoplasmic staining and tighter tangles in the TETC of the corresponding brain, the HIP, and parts of the cortex (FC, TC). These phenomena were similar to those observed in AD patients [49], suggesting that pre-NFTs and eNFTs in the brains of T2DM monkeys represent an early stage of abnormal tau protein processing. In addition, we found progressively activated glial cells exhibiting varied morphological features. Although the PSD95 levels decreased in the brains of T2DM monkeys, no significant change was detected in the intensity of synaptophysin immunopositivity. Similarly, we found no neurodegeneration and neuronal death in Aβ-deposited T2DM monkeys. This result was considered normal, as synapses do not decrease significantly in the early stages of AD, which is consistent with the preclinical AD patients who do not develop significant clinical symptoms [50].

### Limitations

This study has some limitations. As mentioned in the experimental sections, the studies were performed on two batches of monkeys (both batches comprising T2DM monkeys and NC monkeys). To detect AD biomarkers in T2DM monkeys, and due to these monkeys being still alive, cognitive-behavioral evaluation or PET imaging is planned in the future to corroborate that T2DM monkeys can provide richer evidence as a preclinical AD model. Therefore, brain tissue samples from T2DM monkeys already available were used to evaluate AD pathophysiological processes. Despite this, T2DM monkeys were screened using the same fasting glucose criteria. Therefore, although evaluations of the AD biomarker and histopathologic were not performed in the same monkeys, we are positive that the results are conclusive and reliable. The second limitation of the study is a small sample size. Plasma Aβ40 and Aβ42 levels were significantly decreased in T2DM monkeys, but there was only a decreased trend of Aβ42/Aβ40, including CSF, with no reach statistical difference, which can possibly be explained by sample size.

## Conclusions

In conclusion, T2DM monkeys showed elevated levels of peripheral and intracerebral inflammation, positive AD biomarkers in body fluids, and AD-like pathology in the brain, including Aβ and tau pathology, glial cell activation, and partial synaptic damage, but no neuronal degeneration or death, compared to the healthy normal group. Hereby, we consider the T2DM monkeys with elevation of the peripheral pro-inflammatory factor level and positive AD biomarkers can be potentially regarded as a preclinical AD model.

### Electronic supplementary material

Below is the link to the electronic supplementary material.

Supplementary Material 1

Supplementary Material 2

## Data Availability

The datasets supporting the conclusions of this article are included within the article and its additional files.

## Abbreviations

AD: Alzheimer’s disease
Aβ: amyloid-beta
APP: amyloid precursor protein
NFTs: neurofibrillary tangles
CNS: central nervous system
T2DM: type 2 diabetes mellitus
PB: peripheral blood
CSF: cerebrospinal fluid
T231: threonine 231
AT8: serine202/threonine 205
FPG: fasting plasma glucose
ADA: American Diabetes Association
ELISA: enzyme-linked immunosorbent assay
IL-6: Interleukin-6
TNF-α: tumor necrosis factor-α
SII: system immune-inflammation index
Aβ42: β-Amyloid 1–42
Aβ40: β-Amyloid 1–40
NfL: neurofilament light chain
GFAP: glial fibrillary acidic protein
HRP: horseradish peroxidase
DAB: substrate chromogen 3,3’-diaminobenzidine tetrahydrochloride hydrate
FJC: Fluoro-Jade C
ANOVA: analysis of variance
PFC: prefrontal cortex
FC: frontal cortex
TC: temporal cortex
EC: entorhinal cortex
p-tau: phosphorylated tau
TETC: transentorhinal
HIP: hippocampus
eNFTs: extracellular- neurofibrillary tangles
NeuN: neuronal nucleus
